# Transcutaneous Electrical Acupoint Stimulation Improves the Postoperative Quality of Recovery and Analgesia after Gynecological Laparoscopic Surgery: A Randomized Controlled Trial

**DOI:** 10.1155/2015/324360

**Published:** 2015-06-11

**Authors:** Yusheng Yao, Qiuyan Zhao, Cansheng Gong, Yihuan Wu, Ying Chen, Liangcheng Qiu, Xiaodan Wu, Yanqing Chen

**Affiliations:** ^1^Department of Anesthesiology, The Shengli Clinical Medical College of Fujian Medical University and Fujian Provincial Hospital, Fuzhou 350001, China; ^2^Department of Traditional Chinese Medicine, Fujian Provincial Hospital, Fuzhou 350001, China

## Abstract

*Background*. We conducted this prospective, randomized, double-blind, placebo-controlled study to evaluate the effects of transcutaneous electric acupoint stimulation (TEAS) on the quality of recovery (QoR) and postoperative analgesia after gynecological laparoscopic surgery. *Methods*. 74 American Society of Anesthesiologists physical status (ASA) I or II patients undergoing gynecological laparoscopic surgery were randomly allocated to TEAS or control groups. The primary outcome was the quality of recovery, which was assessed on the day before surgery and 24 h after surgery using a 40-item questionnaire. Secondary outcomes included postoperative pain scores, the incidence of postoperative nausea and vomiting (PONV), duration of postanesthesia care unit (PACU) stay, and patient's satisfaction. *Results*. The TEAS group had higher QoR scores than control group upon 24 h after surgery (177 versus 165; *P* < 0.001). Compared with the control group, postoperative pain scores and the cumulative number of opioids administered were lower in the TEAS group patients (*P* = 0.04). TEAS reduced the incidence of PONV and dizziness, as well as duration of PACU stay. Simultaneously, the patient's satisfaction scores were higher in the TEAS group (*P* = 0.002). *Conclusion*. Preoperative TEAS enhances QoR, improves postoperative analgesia and patient's satisfaction, alleviates postoperative side effects, and accelerates discharge after general anesthesia for gynecological laparoscopic surgery.

## 1. Introduction

Gynecological laparoscopy is considered to be a minimally invasive procedure. However, as common complications of anesthesia and surgery, postoperative pain, nausea, and vomiting remain problematic despite use of analgesics and antiemetics. These complications delay the patient's recovery from anesthesia, extend their hospital stay, and increase overall healthcare costs [1, 2]. Due to side effects of drug therapy, an integrated approach combining pharmacological methods and various complementary analgesic techniques has been recommended in clinical practice [3]. These nonpharmacological therapies include acupuncture, transcutaneous electrical nerve stimulation (TENS), and acupressure.

Acupuncture is widely accepted in China, Japan, and Korea, which is considered as a complementary intervention for acute and chronic pain of various origins [4, 5]. In addition, acupuncture is commonly used and recommended as part of a balanced anesthetic technique in the above countries [6, 7]. But, acupuncture is invasive, and its application requires a physician experienced in this technique. TEAS is a form of noninvasive electrical stimulation that produces a perceptible sensation via electrodes attached to the skin. It has no risk of infections and can potentially be applied by medical personnel with minimal training. Clinical trials have demonstrated that TEAS reduces the consumption of intraoperative anesthetics and general anesthesia related side-effects [6, 8, 9]. However, the effect of TEAS on the quality of recovery and postoperative pain in patients undergoing gynecological laparoscopic surgery remains unclear. Therefore, we conducted this prospective, randomized, double-blind study to verify the hypothesis that preoperative TEAS could improve the quality of recovery (QoR) and postoperative analgesia after gynecological laparoscopic surgery.

## 2. Materials and Methods

### 2.1. Patients

This study was a single-center, prospective, randomized, double-blind, placebo-controlled trial. The study protocol was approved by the Institutional Review Board of Fujian Provincial Hospital (Ref. K2014-05-008). We enrolled 74 consecutive subjects, aged 18 to 60 years and American Society of Anesthesiologists physical status I-II, who underwent general anesthesia for elective gynecological laparoscopic surgery from May 2014 to November 2014 at Fujian Provincial Hospital. The exclusion criteria were as follows: potentially difficult airway, sore throat, a history of chronic pain, drug or alcohol abuse, mental disorder, obesity (BMI > 30 kg/m^2^), intake of any analgesic drug within 48 h before surgery, and previous experience with acupuncture treatment. The study was performed in line with the principles of the Declaration of Helsinki and the CONSORT statement. Written informed consent was obtained from all subjects before randomization.

### 2.2. Randomization and Blinding

Patients were assigned to either the TEAS group or the control group by a table of computer-generated random numbers. The allocation ratio was 1 : 1 for the two groups. Group assignments were sealed in sequentially numbered opaque envelopes. The patients, attending anesthesiologist, surgeons, recovery ward nurses, data collectors, and the person who performed the final statistical analysis were blinded to group assignment.

### 2.3. Study Protocol

**Figure 1:**
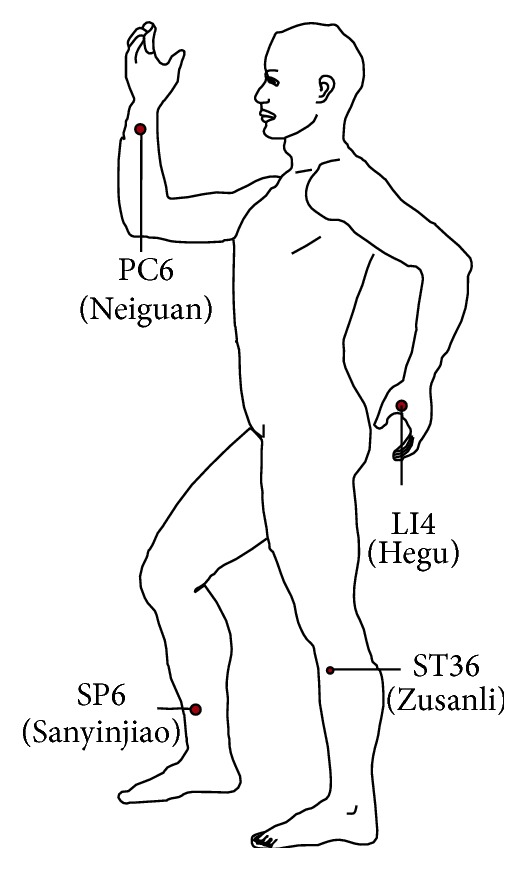
Location of Hegu (LI4), Neiguan (PC6), Zusanli (ST36), and Sanyinjiao (SP6) acupoints.

Patients in the TEAS group received preoperative TEAS for 30 min before the induction of anesthesia in the holding area. TEAS was applied to four pairs of acupoints: bilateral Hegu (LI4), Neiguan (PC6), Zusanli (ST36), and Sanyinjiao (SP6). These acupoints were identified according to the traditional anatomical localisations (Figure 1). TEAS was performed with a dense-disperse frequency of 2/10 Hz and an intensity of 6–9 mA for 30 min using the Hans electronic acupuncture apparatus (HANS-100B, Nanjing Jisheng Medical Technology Company, Nanjing, China). The optimal intensity was adjusted to maintain a slight twitching of the regional muscle according to individual maximum tolerance. In the control group, the patients were connected to the apparatus, but electronic stimulation was not applied.

### 2.4. Standardized Anesthesia

All patients were fasted for at least 8 h and premedicated with IV midazolam 0.05 mg/kg 30 min before anesthesia induction. Standard monitoring, including electrocardiogram, noninvasive blood pressure, pulse oximetry, and temperature, was used in all patients for the duration of surgery. General anesthesia was induced with IV sufentanil 0.5 *μ*g/kg and propofol 2.0 mg/kg. Tracheal intubation was facilitated with cisatracurium 0.15 mg/kg. After intubation, mechanical ventilation was used to maintain P_ET_CO_2_ at 35–45 mmHg. Anesthesia maintenance was achieved with sevoflurane 2%-3% according to both hemodynamic parameters and bispectral index (BIS) of 40–60. Perioperative fluids were administrated in a standardized way, and normothermia (36°C to 37°C) was maintained by a warming device (Bair Hugger; Augustine Medical Inc., Eden Prairie, USA). All patients received IV tropisetron 5 mg 30 min before the end of surgery. Neuromuscular blockade was antagonized using neostigmine 0.02 mg/kg and atropine 0.01 mg/kg.

### 2.5. Study Outcomes

The primary outcome was the quality of recovery, which was assessed on the day before surgery and 24 h after surgery using a 40-item questionnaire (QoR-40) [10]. The global QoR-40 score ranges from 40 to 200, representing extremely poor to excellent quality of recovery, respectively.

Secondary outcomes were postoperative pain scores, the incidence of nausea and vomiting, duration of PACU stay, and patient's satisfaction. Postoperative pain was assessed using Visual Analogue Scale (VAS) in 24 h after surgery. If the VAS score was greater than or equal to 4, the patient received IV sufentanil 0.05 *μ*g/kg as rescue analgesia. Patient's satisfaction was evaluated on postoperative 24 h with a 10-point numerical rating scale: 10 = excellent, 1 = bad. Patient's satisfaction is defined as the scale with a threshold value of greater than or equal to 8.

### 2.6. Statistical Analysis

Our sample size calculation for the two-tailed testing of the TEAS superiority hypothesis was based on the global QoR-40 score. A 10-point difference represents a clinically relevant improvement in quality of recovery based on data from a previous study [10]. A mean (standard deviation) of the QoR score at 24 h postoperative equivalent to 171 (14.3) was estimated based on our pilot study. A power analysis using a type I error estimate of 5% (alpha = 0.05) and a power (1-Beta) of 80% indicated that a sample of 34 subjects per group would be required. Allowing for approximately 10% incomplete follow-up or dropout, a total of 74 subjects were enrolled in this study.

Statistical analysis was performed using SPSS version 18.0 (SPSS Inc., Chicago, IL, USA). The normality of distribution was assessed with the Kolmogorov-Smirnov test. Parametric data were reported as mean (standard deviation (SD)) and analyzed with the independent *t*-test, and nonparametric data were reported as median and (interquartile range (IQR)) and analyzed using the Mann-Whitney *U* test. Categorical variables were reported as the number of patients (%) and evaluated using the *x* ^2^ or Fisher's exact test when appropriate. The level of significance was considered at a *P* value of less than 0.05.

## 3. Results

**Figure 2:**
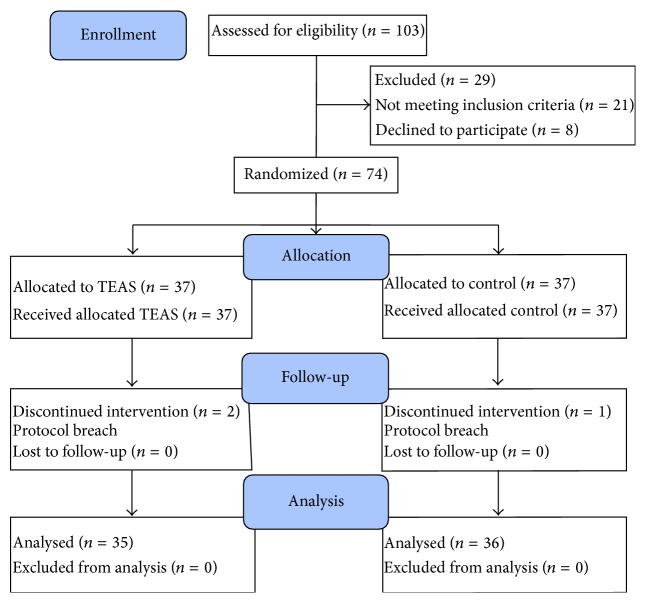
Consolidated Standards of Reporting Trials (CONSORT) flow diagram depicting the progress of subject through the trail. TEAS: transcutaneous electric acupoint stimulation group.

We initially assessed 103 patients for eligibility to participate in this study (Figure 2). Of these, 21 patients did not meet the inclusion criteria, 8 declined to participate, and the remaining 74 patients enrolled to the study. Two patients from the TEAS group and one patient from the Control group were later excluded because of protocol breach. A total of 71 patients randomized to treatment allocation completed the study and their data were included in the analysis. Patient demographic characteristics, type, and durations of procedures were similar with no statistically significant differences (*P* > 0.05) between groups (Table 1).

Patients in the TEAS group had significantly higher QoR scores on 24 h after surgery (*P* < 0.001). As shown in Table 2, the QoR-40 scores (mean (SD)) were 176.5 (10.2) and 164.8 (14.7) in the TEAS group and the control group, respectively. The improvement in QoR scores in the TEAS group reflected improvements in the dimensions of emotional status (3 points), physical comfort (4.5 points), psychological support (3 points), physical independence (1 point), and postoperative pain (2.5 points).

**Figure 3:**
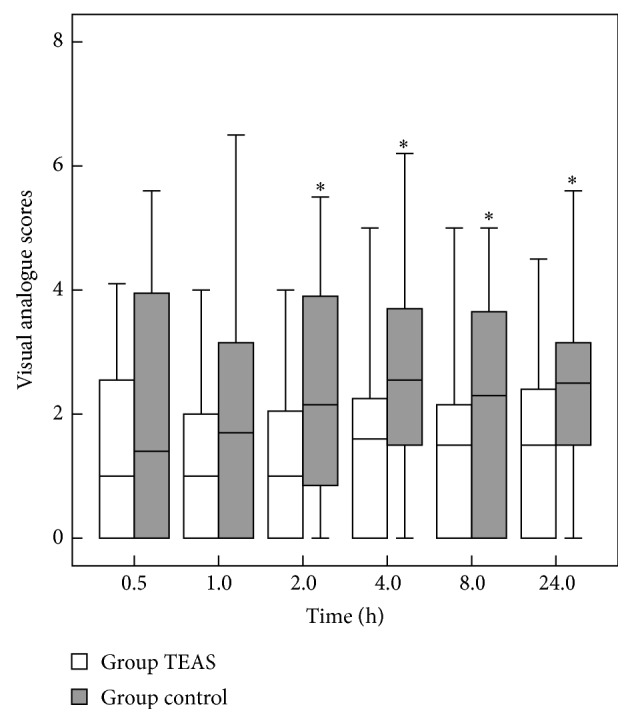
Postoperative visual analogue scores in patients receiving transcutaneous electrical acupoint stimulation (white square) or control treatment (grey square). Error bars are interquartile range. ^*∗*^ *P* < 0.05.

Postoperative pain scores were reduced after TEAS at 0.5 h, 1 h, 2 h, 4 h, 8 h, and 24 h after surgery (Figure 3). Compared with the control group, the cumulative number of opioids administered was significantly lower in the TEAS group patients (*P* = 0.04). In addition, the time to first request of rescue analgesia was longer in the TEAS group (*P* = 0.039).

As shown in Table 3, patients in the TEAS group alleviated the incidence of PONV and dizzy, as well as shortened the duration of PACU stay (7.9 min or 22.1%). Simultaneously, the patient's satisfaction scores were significantly higher in the TEAS group than in the control group (*P* = 0.002).

## 4. Discussion

This study demonstrates that the preoperative TEAS at Hegu (LI4), Neiguan (PC6), Zusanli (ST36), and Sanyinjiao (SP6) enhances the QoR-40 and postoperative analgesia in patients undergoing gynecological laparoscopic surgery. In addition, TEAS reduces the incidence of general anesthesia induced side effects, such as dizziness, nausea, and vomiting, shortens the duration of PACU stay, and improves patient's satisfaction. These results suggest that TEAS may be an interesting complementary and alternative analgesic in human subjects.

In this present study, judging from pain intensity and supplemental analgesic requirement, we revealed that preoperative TEAS is an appropriate procedure for acute postoperative analgesia, which was comparable with the previous studies [11–13]. According to the theory of traditional Chinese medicine, surgery as well as anesthesia breaks the balanced state of the human body and disturbs the movement of both qi and blood. Although several literatures supporting that electroacupuncture inhibits sensory and affective dimensions of pain via activation of endogenous pathways, both by exerting a direct inhibitory effect on opioid-sensitive spinal cord interneurones and by promoting enkephalin release [14–16]. The underlying mechanisms of TEAS's analgesic effects have not been clearly clarified.

The Hegu (LI4) belongs to the Large Intestine Meridian of Hand-Yangming and proved to be associated with analgesic and sedative effect [17]. Stimulation of the Neiguan (PC6), which is one of important acupoints on the Hand-Jueyin pericardium meridian, is suggested to mitigate PONV after surgery [18]. The Zusanli (ST36) is the conjunction point of the stomach channel of the Foot-Yangming. The previous study showed acupuncture at Zusanli (ST36) can improve upper and lower abdominal symptoms induced by rectal distension [19]. Stimulation of the Sanyinjiao (SP6) is effective in relieving labor pain [20] and dysmenorrhea [21]. From our point of view, applying acupuncture to multiple points achieves greater effects than any single one. Therefore, in our current trial, the acupuncture points Hegu (LI4), Neiguan (PC6), Zusanli (ST36), and Sanyinjiao (SP6) were selected. The dense-disperse frequency of 2/10 Hz was chosen based on previous literatures [22, 23]. The duration of TEAS was 30 minutes in this study, which fits our clinical practice.

In addition, our data confirmed that preoperative TEAS attenuates the incidence of PONV and dizziness. Many studies have supported the efficacy of Neiguan (PC6) acupoint stimulation for preventing PONV [24, 25]. The potential benefits for patients, especially in outpatient gynecological laparoscopic surgery, are faster recovery and rapid discharge after procedure.

There are some limitations to this trial that require consideration when interpreting the results. First, the QoR-40 questionnaire we used was initially tested in the Australian population; cultural differences between countries may limit its generalizability [26]. Second, this is a single center study in a strictly defined patient population. This may potentially limit external validity of the findings [27].

In conclusion, this study is the first verification that preoperative TEAS is effective intervention in improving the quality of recovery, postoperative analgesia, and patient's satisfaction and accelerating discharge after general anesthesia for gynecological laparoscopic surgery. Further studies are required to identify the most beneficial use of TEAS time, frequency, and intensity.

## Figures and Tables

**Table 1:** Patient characteristics and operation details.

	Group TEAS (*n* = 35)	Group C (*n* = 36)	*P* value
Age (year)	34.2 (7.2)	35.6 (8.7)	0.47
ASA (I/II)	34/1	34/2	1.0
Height (cm)	159.1 (6.5)	160.4 (4.9)	0.293
Weight (kg)	53.5 (6.7)	55.8 (8.2)	0.209
Type of surgery			0.562
Ovarian cystectomy	30 (85.7%)	29 (80.6%)	
Myomectomy surgery	5 (14.3%)	7 (19.4%)	
Preoperative QoR-40	186.7 (9.9)	184.6 (8.5)	0.339
Duration of surgery (min)	64.5 (9.2)	63.9 (9.3)	0.776
Duration of anesthesia (min)	72.1 (9.5)	72.8 (9.8)	0.791

Values are mean (SD), or number (%). TEAS: transcutaneous electric acupoint stimulation; C: control.

**Table 2:** QoR-40 dimensions and global scores.

	Group TEAS (*n* = 35)	Group C (*n* = 36)	*P* value
QoR-40 dimensions			
Emotional state	40.5 (3.4)	37.9 (5.1)	0.013
Physical comfort	50.8 (4.1)	46.3 (5.7)	<0.001
Psychological support	32.9 (2.3)	31.7 (2.3)	0.029
Physical independence	20.6 (2.4)	19.7 (3.0)	0.152
Pain	31.7 (2.4)	29.2 (3.6)	0.001
Global QoR-40	176.5 (10.2)	164.8 (14.7)	<0.001

Values are mean (SD). TEAS: transcutaneous electric acupoint stimulation; C: control.

**Table 3:** Patient characteristics in 24 h after surgery.

	Group TEAS (*n* = 35)	Group C (*n* = 36)	*P* value
Time to first rescue analgesia (min)	59 (31–1440)	47 (13–196)	0.039
Cumulative number of rescue analgesia	1 (1–3)	3.5 (2–7.8)	0.004
Duration of PACU stay (min)	27.8 (8.3)	35.7 (7.2)	<0.001
Dizziness	14 (40.0%)	28 (77.8%)	0.001
Nausea	17 (48.6%)	26 (72.2%)	0.041
Vomiting	7 (20.0%)	19 (52.8%)	0.004
Patient's satisfaction score	8 (6–8)	6 (5–7)	0.002
Satisfaction score ≥8, *n* (%)	18 (51.4%)	6 (16.7%)	0.002

Values are mean (SD), median (IQR), or number (%). TEAS: transcutaneous electric acupoint stimulation; C: control; PACU: postanesthesia care unit.
